# Neonatal Plasma Polarizes TLR4-Mediated Cytokine Responses towards Low IL-12p70 and High IL-10 Production via Distinct Factors

**DOI:** 10.1371/journal.pone.0033419

**Published:** 2012-03-19

**Authors:** Mirjam E. Belderbos, Ofer Levy, Femke Stalpers, Jan L. Kimpen, Linde Meyaard, Louis Bont

**Affiliations:** 1 Department of Pediatrics, University Medical Center Utrecht, Utrecht, The Netherlands; 2 Division of Infectious Diseases, Children's Hospital Boston and Harvard Medical School, Boston, Massachusetts, United States of America; National Council of Sciences (CONICET), Argentina

## Abstract

Human neonates are highly susceptible to infection, which may be due in part to impaired innate immune function. Neonatal Toll-like receptor (TLR) responses are biased against the generation of pro-inflammatory/Th1-polarizing cytokines, yet the underlying mechanisms are incompletely defined. Here, we demonstrate that neonatal plasma polarizes TLR4-mediated cytokine production. When exposed to cord blood plasma, mononuclear cells (MCs) produced significantly lower TLR4-mediated IL-12p70 and higher IL-10 compared to MC exposed to adult plasma. Suppression by neonatal plasma of TLR4-mediated IL-12p70 production, but not induction of TLR4-mediated IL-10 production, was maintained up to the age of 1 month. Cord blood plasma conferred a similar pattern of MC cytokine responses to TLR3 and TLR8 agonists, demonstrating activity towards both MyD88-dependent and MyD88-independent agonists. The factor causing increased TLR4-mediated IL-10 production by cord blood plasma was heat-labile, lost after protein depletion and independent of lipoprotein binding protein (LBP) or soluble CD14 (sCD14). The factor causing inhibition of TLR4-mediated IL-12p70 production by cord blood plasma was resistant to heat inactivation or protein depletion and was independent of IL-10, vitamin D and prostaglandin E2. In conclusion, human neonatal plasma contains at least two distinct factors that suppress TLR4-mediated IL-12p70 production or induce IL-10 or production. Further identification of these factors will provide insight into the ontogeny of innate immune development and might identify novel targets for the prevention and treatment of neonatal infection.

## Introduction

Toll-like receptors (TLRs) are key pathogen recognition receptors of the innate immune system that recognize highly conserved structures expressed by micro-organisms, called pathogen-associated molecular patterns (PAMPs) [1]. TLR-mediated recognition of microbial products or endogenous “danger” signals results in production of cytokines and chemokines that initiate the innate immune response and instruct the adaptive immune system [2]. The balance between the production of pro- and anti-inflammatory cytokines is tightly regulated, presumably to allow efficient immune responses while protecting the host from excessive inflammation [3]. Birth imposes major challenges on the regulatory capacity of the immune system, including prevention of harmful allo-immune reactions to maternal antigens *in utero*, and balancing the transition from a sterile intra-uterine environment to the microbe-rich outside world [4]. To face these demands, neonatal TLR responses are physiologically biased against the production of pro-inflammatory cytokines [5]–[7]. However, this bias leaves the newborn highly susceptible to infections [8]. Recent studies indicate that despite impaired production of Th1-type cytokines, the neonatal TLR system is not generally depressed. In fact, neonatal innate immune cells, including monocytes and dendritic cells (DC), demonstrate increased TLR-mediated production of certain cytokines (IL-10, IL-23) [9]–[13]. Together, this suggests the existence of regulatory mechanisms that actively polarize the neonatal TLR system towards decreased production of Th1-polarizing cytokines while Th2- and Th17-polarizing responses are relatively preserved. Insight into the mechanisms underlying this polarization is of great interest, as a biological phenomenon and to identify potential targets for the prevention and treatment of neonatal infections.

Despite many studies describing impaired TLR-mediated production of Th1-polarizing cytokines by cord blood innate immune cells [12]–[16], the underlying mechanisms are incompletely characterized [9], [13], [17], [18]. In addition, it is as yet unclear whether the mechanisms that suppress Th1-polarizing cytokine production in cord blood extend into the neonatal and infant age. We have previously demonstrated that whole blood TLR4-mediated cytokine responses at birth and at the age of one month are distinct from adult responses and characterized by decreased production of IL-12p70 and increased production of IL-10 [14]. In the current study, we aimed to determine whether soluble factors in neonatal plasma contribute to distinct neonatal TLR4-mediated production of IL-12p70 and IL-10.

## Materials and Methods

### Ethics statement

The research protocol was approved by the Medical Ethics Committee of the University Medical Centre Utrecht and written informed consent was obtained from parents of all participants.

### Blood

Blood was obtained from healthy newborns participating in an ongoing birth cohort study on the role of neonatal TLR responses in the pathogenesis of respiratory tract infections and asthma [14]. Cord blood was collected immediately after uncomplicated term vaginal delivery, but before delivery of the placenta. Neonatal venous blood was collected from healthy newborns at the age of one month (4–7 weeks, here referred to as ‘neonate’) and adult blood was obtained from healthy volunteers. None of the participants had signs or symptoms of infectious disease, such as respiratory tract complaints or fever, in the two weeks prior to sampling. Blood was collected in sterile, heparin-coated collection tubes (BD Biosciences, Franklin Lakes, NJ). Plasma was prepared within 24 h from blood collection via centrifugation (1000 *g*, 10 min, room temperature), aliquoted into cryovials (Nalge Nunc International, Rochester, NJ) and stored at −20°C prior to use. Pilot experiments demonstrated that times between blood harvesting and plasma preparation from 0 up to 48 h did not affect the modulatory effect of plasma on TLR4-mediated IL-12p70 or IL-10 production (data not shown). MCs were isolated and stimulated immediately after blood collection.

### Immune agonists

*In vitro* stimulation was performed using optimal concentrations of immune agonists and incubation times for cytokine measurements, as titrated in pilot experiments (data not shown). Accordingly, adult peripheral blood mononuclear cells (PBMCs) or cord blood mononuclear cells (CBMCs) were stimulated with polyinosinic∶polycytidylic acid (poly I∶C; 200 µg/mL; Invivogen, San Diego, CA), ultrapure LPS from *E. Coli* (100 ng/mL; InvivoGen), or CL-075 (10 µg/mL, InvivoGen). In cultures using LPS only, no detectable IL-12p70 production was observed. Therefore, for co-stimulation, recombinant IFN-γ was used (20 ng/mL; PeproTech Inc, Rocky Hill, NJ). Addition of IFN- γ did not influence the differential modulation of TLR4-mediated IL-12p70 and IL-10 production by neonatal and adult plasma. Stimulation with IFN- γ only (i.e. without LPS) did not induce any IL-10, IL-12p70 or IL-12p40 in any of the experiments performed (data not shown).

### Cell isolation and stimulation

Adult PBMCs and CMBCs were obtained by Ficoll-Hypaque (Amersham Pharmacia Biotech, Uppsala, Sweden) gradient separation. Cells were washed twice in sterile PBS and seeded in 96-well polystyrene culture plates (Nalge Nunc International, Rochester, NJ) at 1×10^6^/mL in RPMI medium containing 2.5 g/L D-glucose, 1.5 g/L sodium bicarbonate, 1 mM sodium pyruvate, 10 mM HEPES and 300 mg/L L-glutamine (Invitrogen, Breda, NL). MCs were pre-incubated for 30 minutes in plasma prior to addition of TLR agonists. After 24 h incubation at 37°C and 5% CO_2_, supernatants were collected for ELISA, or cells were collected for intracellular cytokine staining. Unless stated otherwise, experiments were performed in 10% plasma.

### Cytokine measurement

Concentrations of IL-12p70 and IL-10 in culture supernatants were determined by ELISA according to manufacturer's instructions (eBioscience, San Diego, CA). Intracellular IL-12p40 production was detected by flow cytometry, using monoclonal antibodies directed against CD14, CD3, CD56, CD16, HLA-DR or, IL-12p40. Intracellular cytokine staining was performed according to manufacturer's protocol, using Golgi-stop to prevent cytokine secretion [19]. Isotype controls were used to correct for non-specific staining. A detailed description of antibody (source, clone, dilution) machine set up, and data acquisition compliant with the MiFlowCyt reporting standards [20] can be found in text S1.

### Plasma heat-inactivation and protein depletion

To characterize the plasma factor responsible for impaired neonatal TLR responses, several approaches were used. Heat-inactivation was performed by heating plasma for 30 min at 56°C. To deplete plasma proteins, plasma was diluted 2-fold in serum-free RPMI medium and boiled for 10 min at 100°C. Afterwards, set precipitate was removed by centrifugation at 13000 *g* for 10 min. To ensure complete depletion/denaturation of plasma proteins, the supernatant was subjected to another cycle of boiling and centrifugation. Efficacy of protein depletion was verified by BCA assay (Pierce, Rockford, IL). As plasma contains several proteins that are needed for LPS-induced cytokine release, all experiments using heat-inactivated or protein-depleted or modified plasma were performed on a background of 5% fetal calf serum (FCS). Addition of FCS up to a concentration of 20% did not affect the differential modulation of LPS-induced cytokine release between cord blood and adult plasma (data not shown).

### IL-10, PGE_2_, vitamin D, sCD14 and LBP

To determine the role of IL-10 as a mediator of TLR4-mediated IL-12p70 production by cord blood plasma, we blocked IL-10 by incubating plasma for 30 min in anti-IL-10 antibody (1 µg/mL, EBioscience, clone JES3-9D7) or isotype control (eBioscience), prior to running the *in vitro* stimulation assay. The effect of PGE_2_ and 1,25-dihydroxy vitamin D (1,25-OHD) on TLR4-mediated cytokine responses was assessed by determining IL-12p70 and IL-10 production in presence of 10% FCS supplemented with recombinant PGE_2_ or 1,25-OHD (both obtained from Sigma Aldrich, Zwijndrecht, The Netherlands). Plasma concentrations of PGE_2_ were measured by ultra sensitive enzyme immunoassay according to manufacturer's instructions (Cayman Chemical, Ann Arbor, MI). Plasma concentrations of 25-OHD (the stable form of vitamin D) were measured with the Modular E170 analyzer (Roche) [21]. Plasma concentrations of sCD14 and LBP were measured by competitive binding assay, according to manufacturer's instructions (Hycult Biotech, Uden, The Netherlands).

### Statistical analyses

Comparisons were made using the Student's t-test. When multiple groups were compared (e.g. age-dependent effects of plasma on TLR4-mediated cytokine responses, concentrations of PGE_2_), one-way ANOVA with post-hoc Bonferroni correction was used. Correlations between TLR4-mediated intracellular IL-12p40 MFI and concentrations of IL-12p70 in culture supernatant and between IL-12p70 production and plasma PGE_2_ concentration were assessed by Pearson correlation. All p-values are two-sided and were considered significant if p<0.05.

## Results

### Differences in TLR4-mediated cytokine production between neonates and adults are due to cellular and soluble factors

**Figure 1:**
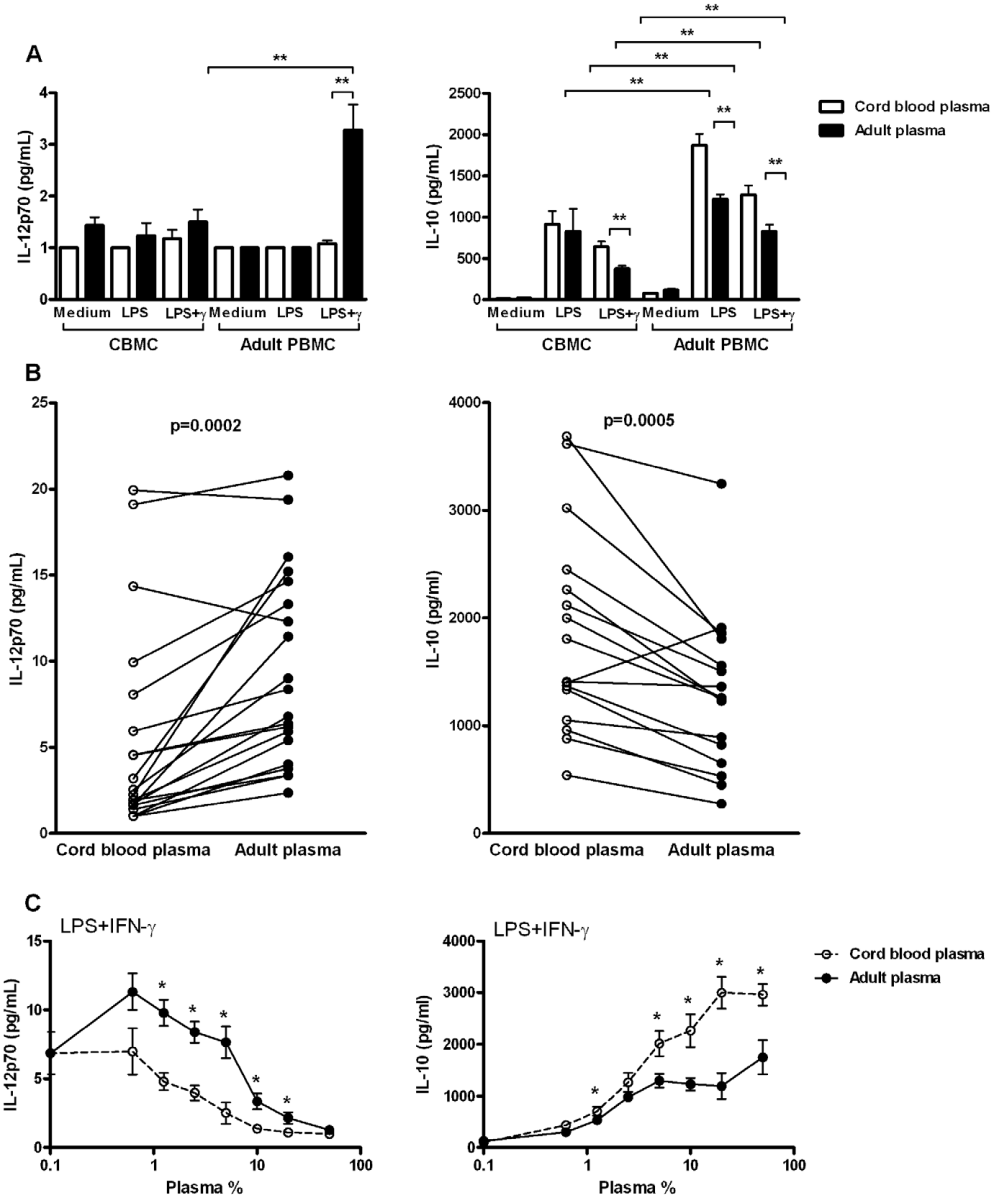
Human plasma suppresses TLR4-mediated production of IL-12p70 while inducing IL-10. A; TLR4-mediated release of IL-12p70 and IL-10 by cord blood mononuclear cells (CBMC) or adult peripheral blood mononuclear cells (PBMC) stimulated in the presence of 10% cord blood plasma or adult plasma. Bars represent mean+SEM of five independent cord blood or adult plasma donors. B; TLR4-mediated release of IL-12p70 and IL-10 by PBMC from three different adult donors stimulated in the presence of 10% cord blood plasma or adult plasma. C; Dose-response curves showing TLR4-mediated release of IL-12p70 and IL-10 by adult PBMC in the presence of increasing concentrations of cord blood plasma or adult plasma. Each dot represents mean ± SEM of five independent cord blood or adult plasma donors. Data are representative of three independent experiments. *; *p*<0.05, **; *p*<0.01.

We and others have previously reported that neonatal TLR responses are distinct from adult responses [5], [11], [14], and that distinct neonatal TLR4-mediated production of IL-12p70 and IL-10 persists up until the age of one month [14]. To determine whether decreased neonatal TLR4-mediated production of IL-12p70 and increased production of IL-10 are due to differences in the cellular or soluble fraction of the blood, we isolated CBMCs and adult PBMCs, resuspended them to equivalent concentrations (1×10^6^) and stimulated them with LPS in the presence of cord blood plasma or adult plasma (Fig. 1). CMBC produced no IL-12p70, independent of the source of plasma (Fig. 1A). Adult PBMCs readily produced IL-12p70, but only when stimulated in the presence of adult plasma, indicating that TLR4-mediated IL-12p70 production is modulated by soluble factors in plasma. TLR4-mediated production of IL-10 by CBMC was also lower than production by PBMC (Fig. 1B). Interestingly, the source of plasma significantly influenced IL-10 production, with increased production in the presence of cord blood plasma by both CBMC and adult PBMC. Together, these findings demonstrate that the differences in whole blood TLR4-mediated production of IL-12p70 and IL-10 between neonates and adults are due to differences in both the cellular and the soluble fraction of the blood.

To further explore modulation of TLR4-mediated cytokine production by soluble factors in plasma, we stimulated PBMCs from twenty (IL-12p70) or sixteen (IL-10) different adult donors in the presence of adult or cord blood plasma (Fig. 1B). Cord blood plasma suppressed TLR4-mediated IL-12p70 production in 18/20 (90%) of PBMC donors (p = 0.0002) and TLR4-mediated IL-10 production was induced by cord blood plasma in 15/16 (94%) of PBMR donors (p = 0.0005).

Potential explanations for the differential modulation of TLR4-mediated cytokine production by cord blood plasma and adult plasma include the lack of factor(s) in cord blood plasma that stimulate LPS+IFN-γ-induced IL-12p70 production (and inhibit IL-10), or the presence of factor(s) that inhibit IL-12p70 (and induce IL-10). To distinguish these possibilities, we added increasing concentrations of cord blood or adult plasma to PBMC cultured in 5% FCS. Addition of either cord blood or adult plasma dose-dependently inhibited TLR4-mediated IL-12p70, while increasing TLR4-mediated IL-10 (Fig. 1C). Inhibition of TLR4-mediated IL-12p70 and induction of IL-10 were more pronounced in the presence of cord blood plasma, with statistically significant stronger polarization of TLR4-mediated cytokine responses compared to adult plasma across plasma concentrations of 1–50% (v/v). Differential modulation of TLR4-mediated IL-12p70 and IL-10 production by cord blood plasma and adult plasma was maintained in the presence of up to 20% FCS (data not shown). These results indicate that human plasma contains one or multiple factors that inhibit TLR4-mediated production of IL-12p70 and/or induce production of IL-10. In addition, they demonstrate that these factors are present at increased concentrations in cord blood plasma.

### Suppression of TLR4-mediated IL-12p70, but not induction of IL-10, by neonatal plasma persists up to the age of one month

**Figure 2:**
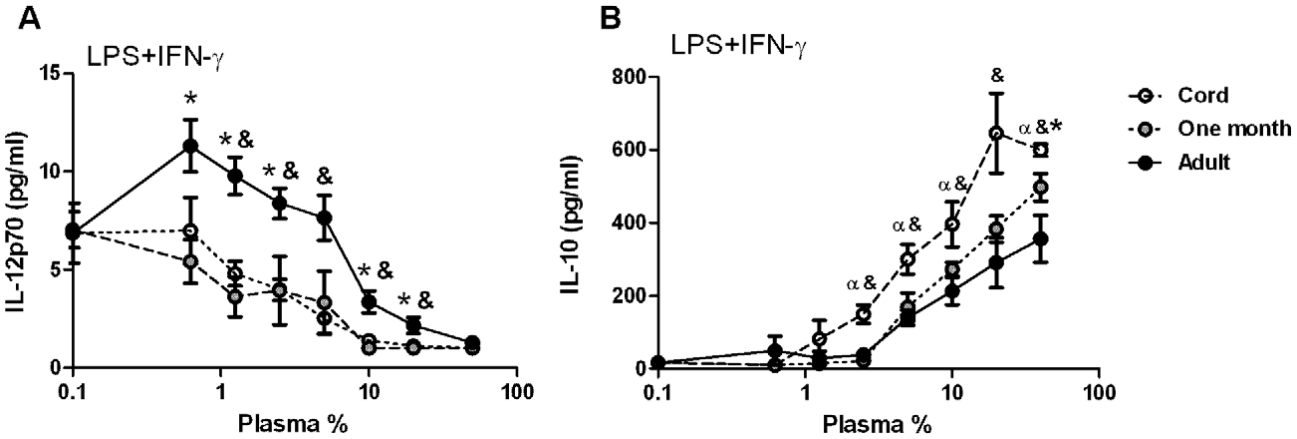
Suppression of TLR4-mediated IL-12p70, but not induction of IL-10, by neonatal plasma is maintained up until the age of one month. Adult PBMC were stimulated with LPS (100 ng/mL) and IFN-γ (20 ng/mL) in the presence of plasma derived from cord blood, healthy newborns aged one month, or adult volunteers. After 24 h incubation, supernatants were collected and concentrations of IL-12p70 (A) and IL-10 (B) were determined by ELISA. Bars represent mean+SEM of three to five different plasma samples. α; *p*<0.05 cord blood vs neonatal plasma, *; *p*<0.05 neonatal vs adult plasma, &; *p*<0.05 cord blood vs adult plasma.

We have previously shown that neonatal whole blood TLR4-mediated cytokine responses are biased towards low IL-12p70 and high IL-10 throughout the first month of life [14]. To determine whether modulation of TLR4-mediated responses by plasma contributes to distinct neonatal cytokine production at the age of one month, we determined TLR4-mediated production of IL-12p70 and IL-10 by adult PBMC stimulated in the presence of plasma derived from cord blood, from healthy neonates aged one month or from adult volunteers. Results showed similar patterns to those previously observed in whole blood (Fig. 2) [14]. TLR4-mediated production of IL-12p70 was significantly decreased in the presence of both cord blood plasma and neonatal plasma obtained at the age of one month compared to adult plasma across plasma concentrations of 1–20% plasma (Fig. 2A). In contrast, TLR4-mediated IL-10 production in presence of plasma of one month old neonates was significantly reduced compared to cord blood plasma and not statistically different from IL-10 production in presence of adult plasma (Fig. 2B). This suggests that TLR4-mediated IL-12p70 and IL-10 are regulated through distinct plasma factors that are differentially present throughout the first month of life.

### Cord blood plasma suppresses TLR4-mediated IL-12p40 production by primary monocytes

**Figure 3:**
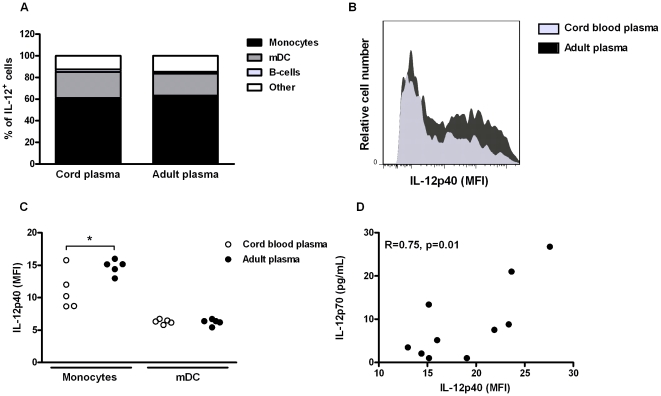
Cord blood plasma suppresses TLR4-mediated IL-12p40 production by primary monocytes. Adult PBMC were stimulated with LPS (100 ng/mL) and IFN-γ (20 ng/mL) in the presence of 10% cord blood plasma or adult plasma. After 24 hours, intracellular levels of IL-12p40 in monocytes (CD14+/HLA-DR+), myeloid dendritic cells (mDC, CD3−/CD16−/CD56−/CD14−/HLA-DR+/CD11c+) and B-cells (CD3−/CD16−/CD56−/CD19+) were determined by flow cytometry. A; Proportion of monocytes, mDC and B-cells among the total population of IL-12p40^+^ cells. Bars represent means of five different plasma samples. B; Representative histograms of monocyte IL-12p40 production upon TLR4-stimulation in the presence of cord blood plasma or adult plasma. C; Mean fluorescence intensity (MFI) of IL-12p40 in monocytes and mDC upon TLR4-stimulation in the presence of cord blood plasma or adult plasma. Each dot represents one individual plasma sample. D; Correlation between TLR4-mediated monocyte IL-12p40 MFI and IL-12p70 protein production by PBMC. Each dot represents one individual plasma sample. All data are representative of three independent experiments. *; *p*<0.05.

Next, we aimed to identify the cell type(s) responsive for the suppressive effect of neonatal plasma on TLR4-mediated IL-12 production by flow cytometry (Fig. 3). Stimulation with LPS+IFN-γ resulted in a strong IL-12p40-positive population (Fig. S1). Among PBMCs, monocytes and mDC were the main producers of TLR4-mediated IL-12p40, accounting for (mean ± SEM) 62.2±1.9 and 22.0±1.8% of IL-12p40 positive cells, respectively (Fig. 3A). Cord blood plasma conferred decreased production of TLR4-mediated IL-12p40 (assessed by mean fluorescent intensity, MFI) by monocytes, but not by mDC (Fig. 3B–C). Although we were unable to detect any IL-12p35, there was a strong correlation (R = 0.75, p<0.01) between the effects of individual plasma samples on monocyte IL-12p40 MFI and PBMC-mediated production of IL-12p70 (Fig. 3D), suggesting that monocyte intracellular IL-12p40 is a reliable marker for extracellular IL-12p70 release. These findings confirm that the majority of TLR4-mediated IL-12p70 is produced by monocytes. Furthermore, they indicate that cord blood plasma suppresses TLR4-mediated IL-12p70 via suppression of monocyte IL-12p40 production.

### Cord blood plasma modulates TLR4-mediated cytokine production through MyD88-dependent and MyD88-independent pathways

**Figure 4:**
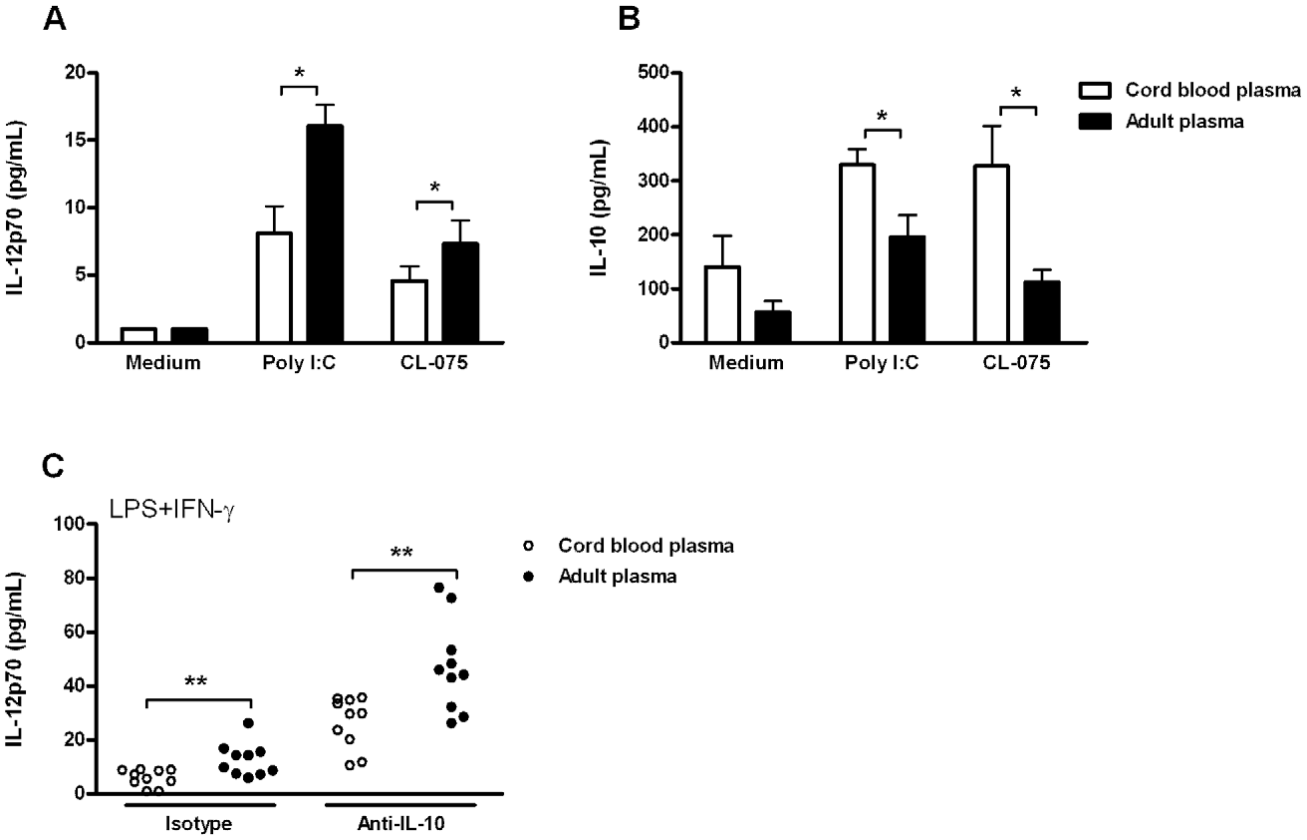
Cord blood plasma polarizes cytokine responses to MyD88-dependent and independent TLR agonists. A–B; Agonist-induced production of IL-12p70 (A) and IL-10 (B) by adult PBMC stimulated in the presence of 10% cord blood plasma or adult plasma. PBMC were stimulated for 24 h with medium, poly I∶C (TLR3, 200 µg/mL) or CL-075 (TLR8, 10 µg/mL). After 24 h, supernatants were collected and cytokine concentrations were determined by ELISA. Bars represent mean+ SEM from five different plasma samples. C; TLR4-mediated production of IL-12p70 by adult PBMC stimulated in the presence of 10% cord blood plasma or adult plasma, with or without IL-10 neutralizing antibody (1 µg/mL). Each dot represents one individual plasma sample. Data are representative of three independent experiments. *; *p*<0.05, **; *p*<0.01.

Among TLRs, TLR4 is unique in its capacity to signal through both MyD88-dependent and MyD88-independent pathways [2]. To determine whether one of these pathways is differentially affected by plasma, we determined the effects of cord blood plasma and adult plasma on responses to TLR3, which signals through MyD88-independent/TRIF dependent pathways, and TLR8, which signals MyD88-dependently (Fig. 4). Cord blood plasma significantly suppressed IL-12p70 production to both agonists (Fig. 4A), while increasing the production of IL-10 (Fig. 4B). This indicates that cord blood plasma polarizes TLR-mediated cytokine production through MyD88-dependent and through MyD88-independent pathways.

To exclude the possibility that suppression of IL-12p70 by cord blood plasma was secondary to increased basal concentrations of IL-10 or to its increased release, we stimulated PBMC in presence of IL-10 neutralizing antibody. Addition of 1 ug/ml IL-10-neutralizing antibody increased TLR4-mediated IL-12p70 production (Fig. 4C), consistent with inhibition of IL-10 signalling. Increased concentrations of anti-IL-10 antibody to 5 ug/ml did not further enhance IL-12p70 production (Fig. S2), indicating that all biologically active IL-10 was effectively inhibited. Inhibition of IL-10 signalling failed to neutralize the difference between cord blood and adult plasma, indicating that suppression of IL-12p70 by cord blood plasma is not mediated via IL-10.

### Plasma regulates TLR4-mediated production of IL-12p70 and IL-10 through distinct factors

**Figure 5:**
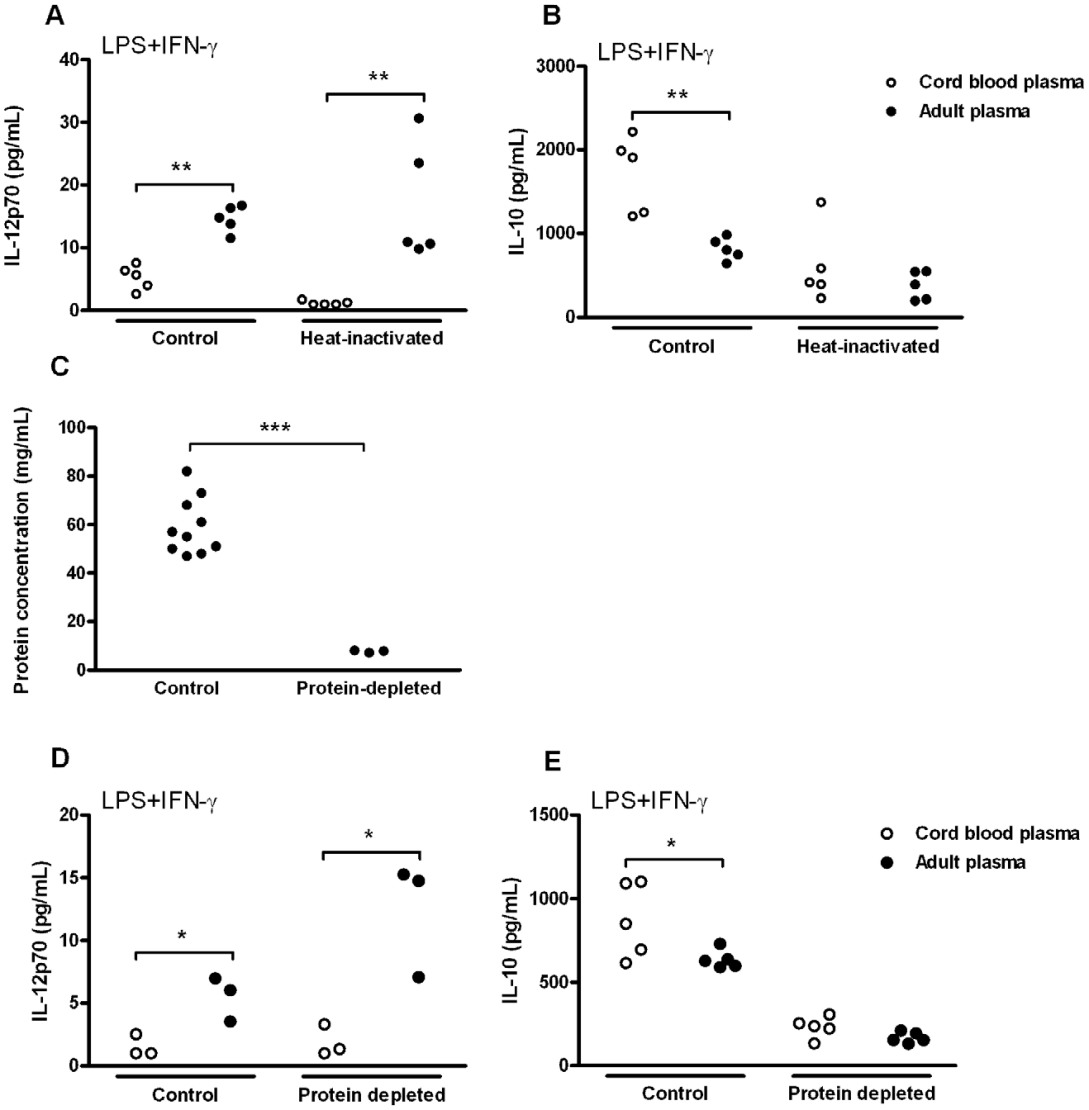
Cord blood plasma modulates TLR4-mediated production of IL-12p70 and IL-10 via distinct factors. A–B; TLR4-mediated production of IL-12p70 (A) and IL-10 (B) by adult PBMC stimulated in the presence of heat-inactivated plasma (30 min at 56°C) or control plasma. C–E; Effect of protein-depleted plasma on TLR4-mediated cytokine responses. Plasma proteins were depleted by two consecutive cycles of boiling and centrifugation and protein concentrations were measured by BCA protein assay (C). Adult PBMC were stimulated with LPS (100 ng/mL) and IFN-γ (20 ng/mL) in the presence of protein-depleted plasma or control plasma, and production of IL-12p70 (D) or IL-10 (E) were measured by ELISA. Each dot represents one individual plasma sample. All data are representative of at least three individual experiments. *; *p*<0.05, **; *p*<0.01, ***; *p*<0.001.

We next aimed to characterize the factor(s) responsible for the polarization of TLR4-mediated cytokine production by cord blood plasma. When assessing the stability of this/these factor(s), we found that heat-inactivation differentially affected TLR4-mediated production of IL-12p70 and IL-10 (Fig. 5). While heat-inactivation did not affect plasma-mediated suppression of IL-12p70 production (Fig. 5A), the differential effect of cord blood plasma and adult plasma on TLR4-mediated IL-10 production disappeared (Fig. 5B). To investigate whether the factor suppressing TLR4-mediated IL-12p70 production is a protein, we depleted plasma proteins by repetitive boiling. This approach resulted in a robust reduction of plasma protein content (mean ± SEM; 59.2±3.7 mg/mL vs 7.8±0.3 mg/mL, p<0.0001)(Fig. 5C) and eliminated the induction of TLR4-mediated IL-10 by cord blood plasma (Fig. 5E). However, protein-depletion of plasma did not abrogate the differential effect of cord blood plasma and adult plasma on TLR4-mediated IL-12p70 production (Fig. 5D). This confirms regulation of TLR4-mediated cytokine production by distinct heat-stable (IL-12p70) and heat labile (IL-10) factors in plasma. Furthermore, failure of protein-depletion to neutralize the differential effect of cord blood plasma and adult plasma on TLR4-mediated IL-12p70 production (Fig. 5D) indicates that the IL-12p70 suppressive factor(s) in cord blood plasma is not a protein.

### The cord blood plasma factors modulating TLR4-mediated IL-12p70 and IL-10 are not PGE_2_, vitamin D, soluble CD14 or LBP

**Figure 6:**
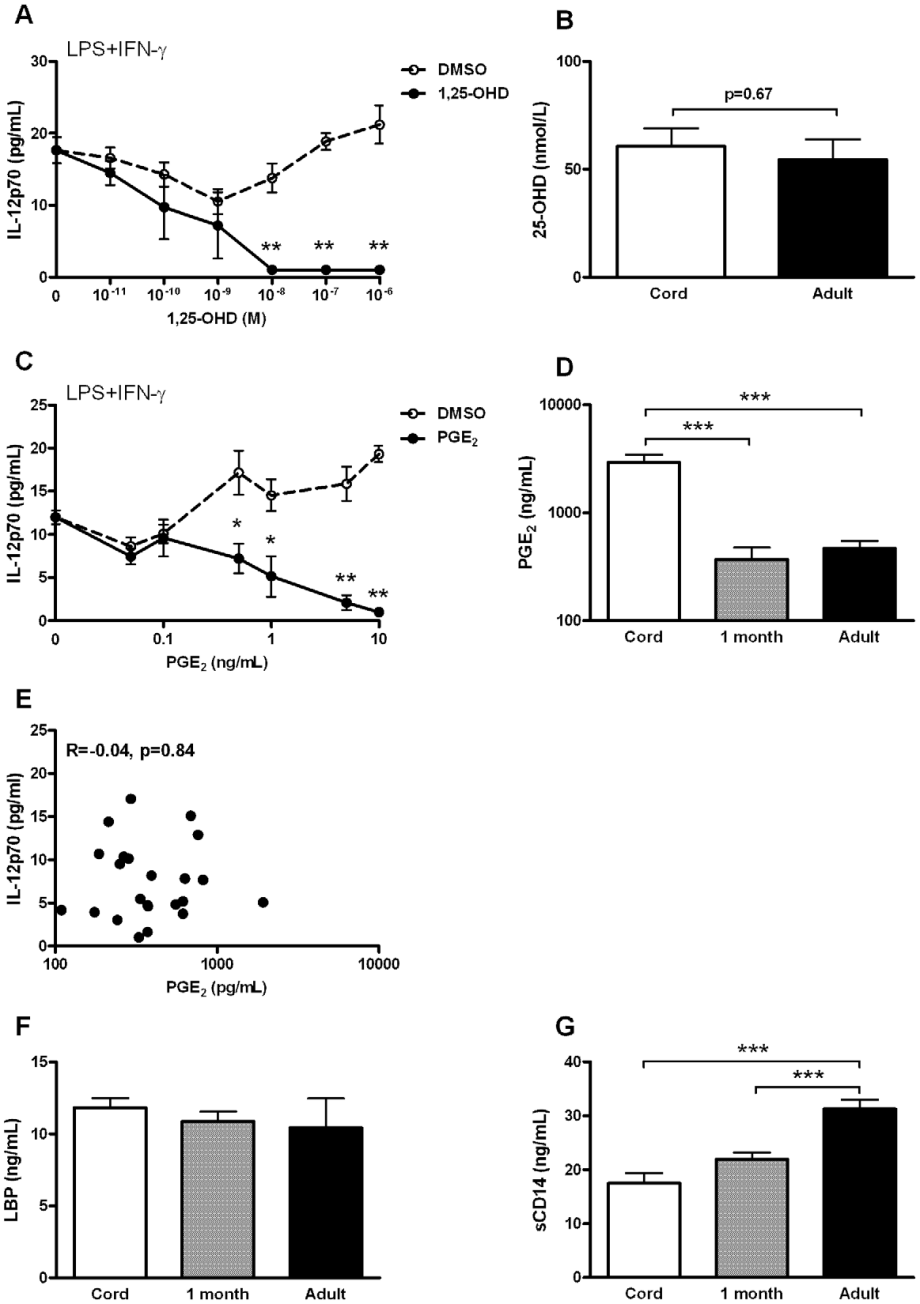
Suppression of TLR4-mediated IL-12p70 and induction of IL-10 by neonatal plasma are not due to prostaglandin E_2_, vitamin D, soluble CD14 or LBP. A; TLR4-mediated production of IL-12p70 by adult PBMC stimulated in the presence of increasing concentrations of 1,25-dihydroxyvitamin D (1,25-OHD). B; Concentrations of 25-hydroxyvitamin D (25-OHD) in cord blood plasma and adult plasma, determined by EIA. C;, TLR4-mediated production of IL-12p70 by adult PBMC stimulated in the presence of increasing concentrations of prostaglandin E_2_ (PGE_2_). D;, Concentrations of PGE_2_ in cord blood plasma and adult plasma. E;, Correlation between plasma concentration of PGE_2_ and its capacity to suppress TLR4-mediated IL-12p70 production. F–G; Concentrations of LBP (F) and sCD14 (G) in cord blood plasma and adult plasma. Data are presented as the mean ± SEM of five to eight individual plasma donors and are representative of three independent experiments. In panel E, each dot represents one individual plasma donor. *; *p*<0.05, **; *p*<0.01; ***; *p*<0.001.

We next aimed to further characterize the factors in neonatal plasma that cause decreased production of TLR4-mediated IL-12p70 and increased IL-10. Vitamin D and PGE_2_ are lipid molecules present in plasma that are known to have IL-12p70 suppressive potential [22], [23]. Indeed, 1,25-OHD (the active form of vitamin D) dose-dependently suppressed TLR4-mediated IL-12p70 production by PBMC (Fig. 6A). At high concentrations, 1,25-OHD also suppressed IL-10 production compared to vehicle control (Fig. S3). However, concentrations of 25-OHD were similar in cord blood plasma and adult plasma (Fig. 6B), suggesting that suppression of TLR4-mediated IL-12p70 by cord blood plasma is not mediated by vitamin D. Similarly, PGE_2_ conferred dose-dependent inhibition of TLR4-mediated IL-12p70 production and at high concentrations, stimulated TLR4-mediated IL-10 production (Fig. 6C and Fig S3). Inhibition of IL-12p70 was observed for concentrations physiologically present in plasma [24], suggesting that plasma PGE_2_ might contribute to suppression of TLR4-mediated IL-12p70 production. However, concentrations of PGE_2_ were elevated in cord blood plasma, but not in plasma from neonates at the age of one month compared to adult plasma (mean ± SEM; 2921±534 pg/mL; 369±105 pg/mL and 469±78 pg/mL, respectively, *p*<0.001) (Fig. 6D). In addition, there was no correlation between PGE_2_ concentrations in individual adult plasma samples and suppression of TLR4-mediated IL-12p70 production (R = −0.02, p = 0.84) (Fig. 6E). Because no TLR4-mediated IL-12p70 was produced in presence of cord blood plasma or plasma from neonates aged one month, no correlations with plasma PGE_2_ concentrations could be calculated for these age groups. Together, these data suggest that PGE_2_ is not the major cord blood plasma factor causing exaggerated suppression of TLR4-mediated IL-12p70 production over adult plasma.

LBP and sCD14 are highly abundant plasma proteins that support cytokine responses (including IL-10) by TLR4 [25]. We hypothesized that increased concentrations of these proteins in neonatal plasma might be responsible for increased TLR4-mediated IL-10 production. However, concentrations of LBP were similar between cord, neonatal and adult plasma (Fig. 6F) and concentrations of sCD14 were even decreased in cord and neonatal plasma compared to adult plasma (Fig. 6G). In addition, we did not observe any correlation between concentrations of LBP or sCD14 and TLR4-mediated IL-10 production (data not shown). Therefore, LBP and sCD14 are not the proteins that cause enhanced TLR4-mediated production of IL-10 in presence of neonatal plasma compared to adult plasma.

## Discussion

Despite increasing awareness of the importance of the TLR system in the neonatal defence against infections [4], much remains to be learned about the mechanisms regulating neonatal TLR responses. Here, we demonstrate that cord blood plasma polarizes TLR-mediated cytokine responses towards low production of IL-12p70 and high production of IL-10. We show that this polarizing effect is present throughout the first month of life, thereby potentially contributing to high neonatal susceptibility to infection. Although to a lesser extent, adult plasma also inhibited TLR4-mediated IL-12p70 production and induced IL-10 production, suggesting that human plasma is a physiologic regulator of TLR responses throughout life.

The strength of our model, namely the use of multiple plasmas per mononuclear cell donor, allowed us to analyze the effect of plasma on TLR responses while eliminating any contribution of cellular differences between neonates and adults. Of note, only adult PBMC (but not CBMC) in the presence of adult plasma were capable of producing IL-12p70 (Fig. 1A), indicating that in addition to the pivotal soluble plasma factors, cellular factors also contribute to the difference in TLR-mediated IL-12 production between neonates and adults.

Previous studies have identified multiple cellular mechanisms that account for different TLR responses between neonates and adults. An elegant study by Goriely et al. demonstrate that decreased TLR4-mediated IL-12p70 production by cord blood dendritic cells is due to impaired nucleosome remodelling, resulting in decreased accessibility of the IL-12p35 promoter [17], [26]. In addition, despite similar basal levels of CD14 [13], neonatal monocytes might express lower levels of MyD88 [26], [27], and demonstrate lower TLR4-mediated p38 phosphorylation [13].

In addition to cellular defects, the current study indicates that differences in the soluble fraction of the blood play a pivotal role in polarization of the neonatal TLR system against Th1-polarizing responses.

Our results demonstrate that polarization of TLR4-mediated cytokine responses by cord blood plasma is not due to a single factor, but mediated by multiple distinct factors that suppress production of IL-12p70 or induce production of IL-10 (Fig. 5). Its resistance to heat-inactivation and protein depletion suggests that the IL-12p70 suppressive factor in cord blood plasma is not a protein. Several non-protein factors in plasma are known to modulate responses by TLR4 and may be responsible for the observed effects in our study.

First, vitamin D is an essential nutrient with multiple immune modulatory functions, including inhibition of IL-12p70 production by dendritic cells [22] and PBMC (Fig. 6). However, concentrations of 25-OHD, the stable form of vitamin D, were similar in cord blood plasma and adult plasma. Thus, although vitamin D may contribute to suppression of TLR4-mediated IL-12p70 by cord blood plasma and adult plasma, it is unlikely to account for the enhanced suppression of TLR4-mediated IL-12p70 by cord blood. Of note, we did not exclude the possibility that increased neonatal conversion of 25-OHD to 1,25-OHD might result in higher plasma concentrations of bioactive vitamin D and increased suppression of TLR4-mediated IL-12p70 production [28].

Second, PGE_2_ is an important lipid mediator that can suppress IL-12p70 production by LPS-stimulated dendritic cells [23]. In figure 6, we confirm that PGE_2_ is a potent suppressor of TLR4-mediated IL-12p70 which is present in increased concentrations in cord blood plasma. However, the observation that PGE_2_ concentrations in plasma from neonates aged one month were similar to adult levels, and the lack of correlation between concentrations of PGE_2_ in individual plasma samples and the extent to which they suppresses TLR4-mediated IL-12p70 production, suggests that PGE_2_ is not the major plasma factor that suppresses neonatal TLR4-mediated IL-12p70 production.

In addition to vitamin D and PGE_2_, plasma contains many other non-protein factors capable of suppressing TLR4-mediated IL-12 production, including metabolites [29], [30], polyunsaturated fatty acids [31] and carbohydrate structures [32]. Selective depletion and separation strategies are needed to identify factors that suppress neonatal TLR4-mediated IL-12p70 production.

In contrast to suppression of TLR4-mediated IL-12p70 production, the factor in cord blood plasma that induces IL-10 is heat-labile and lost upon protein depletion (Fig. 5). A hallmark example of heat-sensitive immune modulatory factors in plasma is the complement family. Although multiple studies have shown that complement factors can suppress TLR-mediated production of pro-inflammatory cytokines [33], [34], their effect on IL-10 is still subject of debate. Whereas C3b and C5a have increase IL-10 production in response to allergen [35] or TLR stimulation [36] respectively, C1q suppresses IL-10 production by human DC [37]. Moreover, neonatal plasma concentrations of complement are diminished, ranging from ∼10–70% of adult levels [38], [39]. Thus, complement is unlikely to account for the enhanced induction of TLR4-mediated IL-10 by cord blood plasma. Other plasma factors capable of enhancing TLR4-mediated IL-10 production include low-density lipoproteins [40], retinoic acid [41], and adenosine [42]. The relative contribution of these and other factors to induction of TLR4-mediated IL-10 by whole plasma will require further investigations.

The suppression of TLR4-mediated Th1-polarizing responses by neonatal plasma also has potential clinical applications. Multiple plasma replacement strategies (e.g. dialysis, filtration) are clinically approved for the treatment of neonatal kidney failure. We hypothesize that plasma may provide a highly interesting target for immune modulation strategies aimed at treating neonatal infection.

In summary, we demonstrate that human newborn plasma contains increased concentrations of distinct soluble factors that suppress IL-12p70 or increase IL-10 production to various TLR agonists, thereby potentially contributing to compromised neonatal innate immune responses. In addition, the concentration-dependent polarization of TLR responses by adult plasma suggests that plasma might contain multiple physiologic regulators of TLR responses throughout life. Further identification of these plasma factors and the mechanism by which they mediate their effect might provide novel therapeutic targets to promote immune responses in case of infection, to enhance responses to vaccination, and to limit excessive inflammation in patients with auto-immune disease.

## Supporting Information

Figure S1 **Flow cytometric detection of TLR4-mediated IL-12p40.** PBMC were stimulated for 4 h with LPS (100 ng/mL) and IFN-γ (20 ng/mL) in the presence of exocytosis inhibitor. Intracellular IL-12p40 was determined by flow cytometry, as described in text S1. Panels demonstrate intracellular IL-12p40 by PBMC, in unstimulated control cultures, LPS+IFN-γ stimulated isotype control stainings, and in LPS+IFN- γ stimulated IL-12p40 stained settings.
(TIF)

Figure S2 **Antibody-mediated inhibition of IL-10.** PBMC were incubated with IL-10 blocking antibody (1 µg/mL or 5 µg/mL) for 30 minutes, and subsequently stimulated with LPS+IFNγ. After 24 hours, culture supernatants were collected and concentrations of IL-12p70 were measured by ELISA. **; p<0.01.
(TIF)

Figure S3 **Effect of vitamin D and PGE_2_ on TLR-4 mediated production of IL-10.** TLR4-mediated production of IL-10 by adult PBMC stimulated in the presence of increasing concentrations of 1,25-dihydroxyvitamin D (1,25-OHD, panel A), or PGE_2_ (panel B). *; *p*<0.05, **; *p*<0.01.
(TIF)

Text S1 **MIFlowCyt standard compliant information for submitted flow cytometric data.**
(DOC)
