# Supplementary material for: Neonatal Plasma Polarizes TLR4-Mediated Cytokine Responses towards Low IL-12p70 and High IL-10 Production via Distinct Factors
Source: PLoS One. 2012 Mar 19;7(3):e33419. doi: 10.1371/journal.pone.0033419 (PMC3307729; doi:10.1371/journal.pone.0033419)
Supplement: Text S1 — MIFlowCyt standard compliant information for submitted flow cytometric data. (DOC) [file pone.0033419.s004.doc]

**Text S1: MIFlowCyt standard compliant information for submitted flow cytometric data.**

**1. Experiment overview.**

**1.1. Purpose**: The purpose of this experiment was to determine whether PBMC stimulated in the presence of neonatal plasma produce less TLR4-mediated IL-12p40 compared to PBMC stimulated in the presence of adult plasma. As a secondary purpose, we aimed to identify the cell type among PBMC that is responsible for TLR4-mediated IL-12 production.

**1.2. Keywords:**

**1.3. Organization:**

**1.3.1.** Meyaard Lab, Utrecht University, NL.

**1.3.2.** Dept of Immunology, University Medical Center Utrecht, PO Box 85090, 3508 AB, Utrecht, Netherlands.

**1.4. Primary Contact:**

**1.4.1.** P.I. Prof. Dr. L. Meyaard, l.meyaard@umcutrecht.nl

**1.4.2.** PhD student. Drs. M. Belderbos m.belderbos@umcutrecht.nl

**1.4.3.** Research Assistant: F. Stalpers, f.stalpers@umcutrecht.nl

**1.5. Date:** Experiments were set up from 01-01-2011 to 01-02-2011 and stained from 01-02-2011 to 01-06-2011.

**1.6. Conclusions:**

Among PBMCs, monocytes and mDC were the main producers of TLR4-mediated IL-12p40, accounting for (mean ± SEM 62.2 ± 1.9 and 22.0 ± 1.8% of IL-12p40 positive cells, respectively (Fig 3A). Cord blood plasma conferred decreased production of TLR4-mediated IL-12p40 (assessed by mean fluorescent intensity, MFI) by monocytes, but not by mDC (Fig 3B-C). Although we were unable to detect any IL-12p35, there was a strong correlation (R=0.75, *p*<0.01) between the effects of individual plasma samples on monocyte IL-12p40 MFI and PBMC-mediated production of IL-12p70 (Fig 3D), suggesting that monocyte intracellular IL-12p40 is a reliable marker for extracellular IL- 12p70 release. These findings confirm that the majority of TLR4-mediated IL-12p70 is produced by monocytes. Furthermore, they indicate that cord blood plasma suppresses TLR4-mediated IL-12p70 via suppression of monocyte IL-12p40 production.

**1.7. Quality Control Measures:**

Unstimulated controls were set up for each condition tested. Single stain controls were included in each experiment using 3 ul of each fluorochrome. Isotype antibodies were used to correct for aspecific staining.

**2. Flow Sample/Specimen Description**

**2.1. Sample/Specimen Material**

**2.1.1. Biological Samples:**

**2.1.1.1. Biological Sample Name**

Adult peripheral blood mononuclear cells

**2.1.1.2.Biological Sample Source:** Healthy human adult peripheral blood mononuclear cells, obtained by Ficoll-Hypaque separation of adult whole blood directly after blood collection.

**2.1.1.2.1. Biological Sample Source Organism:**

**2.1.1.2.1.1. Taxonomy:**

Homo Subspecies sapiens

**2.1.1.2.1.2. Age:**

Adult

**2.1.1.2.1.3. Gender:**

Male and Female.

**2.1.1.2.1.4. Phenotype:**

Healthy (none).

**2.1.1.2.1.5. Genotype:**

Not Applicable.

**2.1.1.2.1.6. Treatment:**

PBMCs were isolated from peripheral blood using Ficoll gradient centrifugation. After purification, cells were washed twice with DPBS then resuspended at 1.0x10^6^ cells/ml in RPMI supplemented with 10% human cord blood plasma or adult plasma. PBMC were then stimulated with LPS (100 ng/ml) and IFN-γ (20 ng/mL) in the presence of Golgi-Plug. After 4 hours, intracellular staining for IL-12p40 was performed.

**2.1.2. Environmental Samples:** Not Applicable.

**2.1.3. Control Sample Description:**

Unstained samples were used as controls to gate live and dead cells. Single stain controls were set up by staining PBMC fractions with optimal concentrations of antibody for each fluorochrome, as titrated in pilot experiments. Isotype controls were used to correct for aspecific staining.

**2.1.4 Sample Treatment Description**

Cells were plated in a 96-well plate at a density of 1x10^6^/ml. Cells were pre-incubated for 30 min in 10% cord blood plasma or adult plasma. Cells were subsequently stimulated with LPS+IFN-γ for 4 hours, in the presence of Golgi-Plug. After 4 hours, intracellular staining was performed for IL-12p40.

**3. Fluorescence Reagent Description**

Fluorescence-labeled antibodies used for flow cytometry are depicted in table 1.

**Table 1:** Antibodies used for intracellular flow cytometry.

| **Epitope** | **Fluorochrome** | **Manufacturer** | **Clone** |
| --- | --- | --- | --- |
| CD3 | PE | Biolegend | UCHT1 |
| CD11c | PE-Cy7 | eBioscience | 3.9 |
| CD14 | PerCP-Cy5.5 | Biolegend | HCD14 |
| CD16 | PE | Sanquin Reagents | CLB-FcR gran/1, 5D2 |
| CD19 | Pacific blue | Biolegend | HIB19 |
| CD56 | PE | Sanquin | NKI-nbl-1 B159 |
| HLA-DR | FITC | eBioscience | LN3 |
| IL-12p40 | APC | BD Biosciences | C11.5 |

**Instrument Details:**

**3.1. Manufacturer:**

BD Biosciences

**3.2. Model:**

BD LSR II 4 Laser, Blue/Red/Violet/UV cat # 347545

**3.3. Instrument Configuration and Settings:**

All lasers, filters and mirrors were manufactured by BD Biosciences.

**3.3.1. Light Sources:**

The light path, filters and detectors are described below in Table 2. The lasers are listed in the order the cells pass through them. The detectors and filters are listed in the order the light hits them, with the exception of FSC which is measured from light that passes through the cell/bead while all the other 488 nm detectors detect light that has been scattered 90°, in the order listed. For example, for blue laser detector A light passes through or is reflected off of filter 1, 735 LP, then the light passes through filter 2, 780/60 BP, then it hits the PMT detector. Light that is reflected off the long pass goes to detector B and so on. For parameters used in this experiment, it is indicated whether Area (-A), Height (-H) or Width (-W) was used. Abbreviations: PMT = photomultiplier tube, PD = photodiode, BP = band pass filter, first number is center of interval, second number is the width of the interval.LP = long pass filter, lets light waves through that have a longer wavelength than the number specified. All LP filters are dichroic and reflect at an angle of incidence at 11.25°. Laser properties and instrument settings are depicted in table 2.

**Table 2:** Lasers used for intracellular flow cytometry

|  | **Detector name (type)** | **Filter 1** | **Filter 2** | **Parameter detected** | **Detector voltage** | **Amplification type** |
| --- | --- | --- | --- | --- | --- | --- |
| **Blue laser (488 nm)** | FSC (PD) | 488/10 BP | Na | FSC-A | 440 | Linear |
| Solid state coherent sapphire blue lased 20 mW | 488 A (PMT) | 735 LP | 780/60 BP | PE-Cy7-A | 555 | LOG |
|  | 488 B (PMT) | 695 LP | 795/40 BP | PERCP, PE-Cy5, PerCP-Cy5.5-A | 510 | LOG |
|  | 488 C (PMT) | 550 LP | 575/26 BP | PE | 418 | LOG |
|  | 488 D (PMT) | 505 LP | 530/30 BP | FITC | 440 | LOG |
|  | 488 G (PD) | blank | 488/10 BP | SSC-A | 330 | Linear |
|  | 488 H (PMT) | blank | na | blank | na |  |
| **Violet Laser (405 nm)** | 405 A (PMT) | 505 LP | 525/50 BP | AmCyan-A, BD Horizon V500 | na |  |
| Coherent VioFlame PLUS laser 25mw | 405 B (PMT) | blank | 440/40 BP | Pacific Blue-A, BD Horizon V450 | 440 | LOG |
|  |  |  |  |  |  |  |
| **Red Laser (637 nm)** | 637 A (PMT) | 735 LP | 780/60 BP | APC-Cy7-A | na |  |
| Solid State Coherent laser 25 mW | 637 B (PMT) | 660/20 BP | na | APC-A | 585 | LOG |

**4. Data Analysis**

**4.1. FCS Data File:**

To request raw data please contact Dr. M. Belderbos: m.belderbos@umcutrecht.nl

**4.1.1. Total Count of Events:** Recorded within individual FCS files, 100 000 events per tube.

**4.2. Compensation Description:**

Compensations were done on acquisition using single stains for each fluorochrome used.

**4.3. Gating (Data Filtering) Description:**

**4.3.1. Gating Information:**

From the total PBMC population, monocytes and lymphocytes were gated based on forward/sideward scatter properties. Within this population, monocytes were identified as CD14^+^/HLA-DR^+^, mDC were identified as CD3^-^/CD16^-^/CD56^-^/CD14^-^/HLA-DR^+^/CD11c^+^, and B-cells were identified as CD3^-^/CD16^-^/CD56^-^/CD19^+^.
